# Improving Corrosion and Photocatalytic Properties of Composite Oxide Layer Fabricated by Plasma Electrolytic Oxidation with NaAlO_2_

**DOI:** 10.3390/ma15207055

**Published:** 2022-10-11

**Authors:** Siti Fatimah, Nisa Nashrah, Kadir Tekin, Young Gun Ko

**Affiliations:** Materials Electrochemistry Group, School of Materials Science and Engineering, Yeungnam University, Gyeongsan 38541, Korea

**Keywords:** pure titanium, plasma electrolytic oxidation, anti-corrosive coating, corrosion, photocatalysis

## Abstract

The present work dealt with the development of a protective and functional oxide layer via one-step plasma electrolytic oxidation (PEO) on pure titanium by employing highly concentrated aluminate solution in a short processing time. A compositional analysis showed that Al_2_TiO_5_ active compound was formed successfully by means of Al_2_O_3_ incorporation when TiO_2_ was spontaneously developed with the aid of plasma swarms. The electrochemical performance showed the protective and functional capabilities of the layer, which was attributed to the respective amounts of Al_2_O_3_ and Al_2_TiO_5_. Such capabilities were achieved in a short processing time, thus reducing the total production cost.

## 1. Introduction

Titanium (Ti) and its alloys have been used for wide applications in the aerospace, automotive, military, energy, and biomedical fields due to their high strength-to-weight ratio, good mechanical behavior, superior biocompatibility, and appreciable corrosion resistance [1,2,3,4,5]. The corrosion resistance of Ti arises from the presence of a naturally occurring thin film on its surface [1,6]. The low thickness, however, inhibits its long-term protection ability owing to its easy destruction in a harsh environment [1,6,7]. The formation of thick and hard layers, therefore, would be desired to eliminate such disadvantages.

Plasma electrolytic oxidation (PEO) is one of the most reliable electrochemical treatments to produce a thick and adherent oxide layer by reforming the surface of valve metals (Al, Mg, Ti, etc.) via a plasma-assisted electrochemical process utilizing high current potential [1,8]. High energy consumption, however, limits its commercial application [6]. On the other hand, the selection of a proper electrolyte solution is pivotal in producing the desired oxide layer via PEO. Among the most highly used alkaline electrolytes for PEO are silicate, phosphate, and aluminate-based solutions [1,9].

A study by Molaei et al. [10] documented that by increasing the NaAlO_2_ concentration from 6 to 10 g/L, it increased the coating compactness, which resulted in an increase of corrosion resistance by up to one-order-of-magnitude. In a report by Cheng et al. [11], the use of concentrated NaAlO_2_ electrolyte (56 g/L) on Al-Cu-Li alloy via PE successfully produced a thick and hard oxide layer with a surprisingly lower electricity cost due to the short processing time as compared to that produced in diluted solution (5 g/L). The concentration of the aluminate electrolyte plays an important role in the porousness of the resultant coating microstructures. This phenomenon can be explained by an appreciable contribution of anion deposition on the substrate surface during coating formation in the concentrated electrolytes. More interestingly, Yang et al. [12] found that a concentrated aluminate solution (20 g/L in phosphate-based electrolyte) had a major impact, assisting in the passivation of the metal substrate during PEO on non-valve metals (Cu, Fe, Ni, etc.). By default, it is kinetically unfavorable for non-valve metals to grow an oxide layer without being dominated by the dissolution of metal ions into the solution during PEO. To satisfy the requirement for the oxidation of such metals, the concentration of hydrogen should be extremely low whilst that of the competitor, i.e., electrolyte species, should be high. The results suggested that an increase in aluminate concentration is favorable for the formation of thick coating with relatively low energy consumption. However, to date, extreme concentrations of aluminate have not been utilized for the PEO process on Ti and its alloys.

Whilst TiO_2_ powder was a popular catalyst in pollutant disposal including sewage treatment and gas purification [13], the combination of an Al_2_O_3_—TiO_2_ layer was reported to exhibit catalytic properties for organic dye degradation [14,15,16]. TiO_2_ powder, however, was unlikely to agglomerate, which caused the active ingredients to be washed away by during the exchange process. In addition, the long process to get the final product has hindered the industrial application of this process. A TiO_2_ oxide layer with high structural reliability, produced by PEO, is required to satisfy practical photocatalysis applications. The present work attempted to scrutinize the effect of high concentrated aluminate solution on the microstructures, electrochemical performance, and catalytic behavior of the resultant TiO_2_-Al_2_O_3_ layer by taking the microstructural evolution and constituent compounds of the oxide layer into account.

## 2. Experimental Procedure

A commercially pure titanium (Ti grade 2) sheet was cut into dimensions of 20 mm × 20 mm × 2 mm and polished with #1200 grit SiC paper prior to PEO. Two different electrolyte systems were prepared from 5 gL^−1^ NaAlO_2_ and 5 gL^−1^ KOH, referred to as diluted electrolyte (bath A), whereas 100 gL^−1^ NaAlO_2_ and 5 gL^−1^ KOH is referred to as concentrated electrolyte (bath B). The values of electrolyte conductivity at 298 K were approximately 23.7 and 150.3 mS·cm^−1^ for bath A and bath B, respectively. The electrolyte temperature was modulated/maintained at 298 K by a water chilling system. PEO was performed under an alternating current with a frequency of 60 Hz and a current density of 100 mA cm^−2^, respectively. PEO was performed for 10 min in bath A and 2 min in bath B during the optimization stage in a bid to obtain the same coating thickness. The coating morphologies were observed by scanning electron microscopy (Hitachi S-4800, Japan). The surface roughness of the samples was measured by atomic force microscopy (AFM, Park systems NX-10, Japan). The constitutive compounds of the oxide layer were examined by X-ray diffraction (XRD, RIGAKU, D-MAX 2500, Japan) with a step size of 0.05 and a scan range from 30° to 90°. Corrosion protection was measured by polarization tests utilizing an electrochemical workstation (Gamry Interface-1000, USA) in a 3.5 wt.% NaCl with details provided in Ref. [17]. The photocatalytic activity responses were monitored by degrading 10 mL methylene blue (MB) dyes with the concentration of 10 ppm under UV-Vis light irradiations with a Xenon lamp of 300 W on the surface of the samples with surface area of 20 mm × 20 mm (one-side of the samples, while the other side was covered by a rubber coating. The degradation/decomposition of the MB dyes solution was monitored through UV–Vis spectrophotometer (Perkin Elmer λ-35, USA) by measuring the absorbance in the wavelength range from 400 to 800 nm. The decrease in absorption was calculated by the formula shown in Equation (1).

(1) $$\mathrm{Degradation}\;\mathrm{rate}=\frac{\left[C_{0}-C_{t}\right]}{C_{0}}$$

The quantitative analysis of MB degradation was calculated using the pseudo-first-order model shown in Equation (s), where *C_e_* is the concentration at equilibrium and *C_t_* is the concentration at the time “*t*”.

## 3. Results and Discussion

### 3.1. Transient Voltage Response and Initial Stage of Plasma Discharges

**Figure 1:**
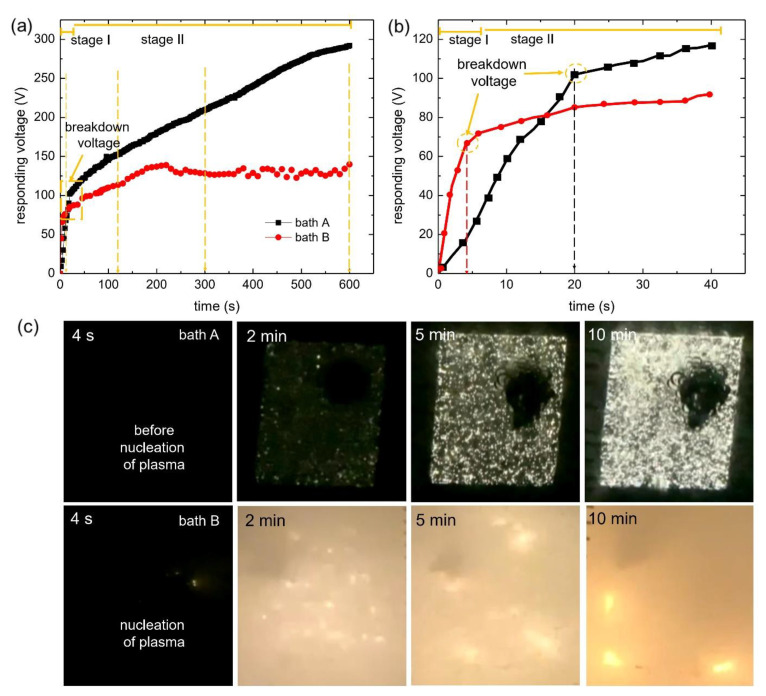
(**a**) Responding voltage vs. coating time curves of the samples treated in in (**a**) low concentration (bath A), and high concentration (bath B) aluminate solution. (**b**) High magnification of (**a**) showing breakdown of the passive film at different voltage and coating time. (**c**) Evolution of plasma discharges formed on the surface of Cp-Ti with respect to coating time in bath A and bath B.

The initiation and the characteristics of plasma discharges, i.e., their size, density, duration, and intensity control the overall microstructure of the oxide layer via PEO. Monitoring the plasma characteristics during PEO is important to understand both the final microstructure of the oxide layer and the incorporation mechanism of the electrolyte species. Figure 1a shows the rms voltage responses of the samples in two different systems, i.e., diluted and highly concentrated electrolytes, ascribed as bath A and bath B, respectively. Figure 1b is a high magnification of Figure 1a, showing the breakdown voltages of both samples.

Irrespective of the electrolyte composition, two different stages were identified from Figure 1a based on the rate of voltage increment with respect to the processing time. In stage I, the voltage increased rapidly following Ohm’s law due to the rapid passivation of the metal substrate, followed by an increase of electrical resistance. The film continued to grow until the breakdown voltage was exceeded, at which point the barrier layer became conductive and the barrier structure of the film became porous, as represented by the gradual decrease of the slope, denoted as stage II. The onset of stage II was preceded by the appearance of tiny, rapid sparks on the sample surface, accompanied by acoustic emissions as a consequence of the breakdown of O_2_ gas.

As shown in Figure 1b, the breakdown voltage was higher for bath A (~100 V) as compared to bath B (~63 V); this was strongly associated with electrolyte conductivity. As documented earlier, the low conductivity of the electrolyte would likely induce higher breakdown voltage, while high conductivity would facilitate a lower breakdown voltage [17,18]. This was because the breakdown voltage was inversely proportional to the conductivity of the electrolyte solution, as suggested by Ikonopisov [19]. It is also worth mentioning that the time to reach the breakdown voltage was faster when a highly concentrated electrolyte was utilized. The time to reach the breakdown was recorded at 20 s for bath A, whilst it was only 4 s for bath B. Considering the voltage and time to reach breakdown, B would likely consume less energy.

The voltage response was relatively unstable in bath B, whereas bath A showed a steady-state behavior. The fluctuating behavior in stage II of bath B might be correlated to the instability of the electrical double layer that is responsible for maintaining the continuous plasma discharges during PEO [20]. As such, this was highly correlated with the plasma behavior during PEO, as recorded in optical images.

**Figure 2:**
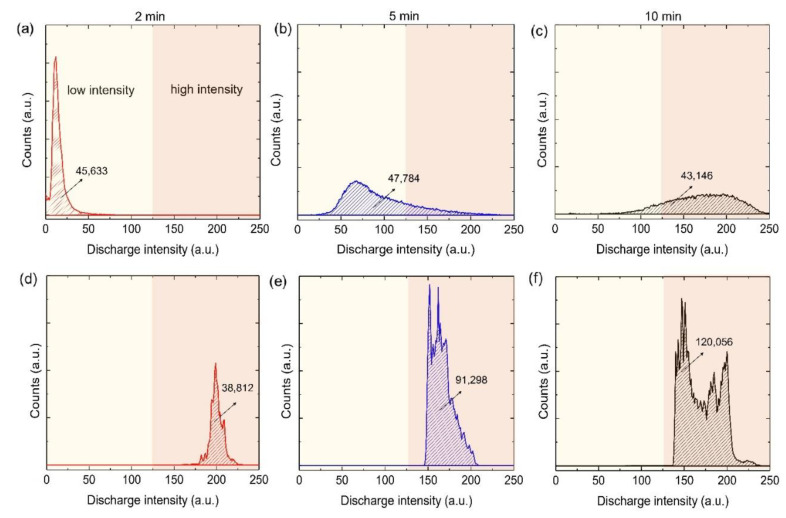
Curves showing the fraction and intensity of plasma discharges during PEO in (**a**–**c**) bath A and (**d**–**f**) bath B.

Figure 1c shows optical images of plasma discharges during PEO in bath A (top) and bath B (bottom). The upper side showed relatively homogeneous plasma discharges whereas the bottom side showed localized plasma discharges with relatively high intensity. The homogeneous plasma discharges might represent the steady voltage response, while local discharges might cause its fluctuation [1]. Regarding the plasma intensity, bath A showed relatively more homogeneous and milder intensity of discharges at the early and middle stages of PEO, as revealed by Figure 2a–c. Meanwhile, bath B was likely to generate inhomogeneous distribution (mainly in high intensity region) and high-intensity discharges (Figure 2d,e).

At low coating time in bath A, low intensity plasma discharges dominated the surface of the substrate, as can be seen from the sharp shape of the curve in the low intensity region. With increasing coating time, higher intensity plasma discharges were formed in bath A, but the majority of plasma discharges were in the low intensity region. Such a phenomenon was due to the increase of plasma size while the coverage remained constant, obeying a postulate by Ikonopisov et al. [19]. In general, the size of plasma discharges was proportional to the plasma temperature or intensity [19]. At longer coating time, a bell-shaped like curve was identified with major distribution in the middle and high intensity regions.

When PEO was prolonged to 10 min, both samples showed an increase in plasma intensity, where bath B was found to have a higher number of plasma discharges and intensity, as can be seen from Figure 2c,f. Moreover, such characteristics might also be correlated with the features of its oxide layer, such as porosity and composition. Based on our video imaging analysis, the high intensity of plasma discharges that developed during the 5- and 10-min PEO processes in bath B might make them unsuitable for obtaining a compact oxide layer due to the destructive behavior of such discharges. On the other hand, the distribution of plasma intensity in bath B processed for 2 min showed a comparable tendency to that of bath A, as shown in Figure 2c,d. The area under the curve for the bath B sample processed in 2 min showed similarity with those found in Figure 2a–c, which indicated that a typical PEO process would be populated by a number of plasma discharges. On the other hand, the area under the curves in Figure 2e-f showed significant increase in the amount of plasma discharges owing to the high plasma intensity covering the whole surface of the anode and illuminating the surrounding solution, leaving no dark spots on the surface. As a result, the video imaging captured a large number of plasma discharges compared to that of bath A [6,19].

From the pre-experimental conditions in the optimization stage, it was found that an identical coating thickness could only be achieved from the sample processed in bath A for 10 min and that processed in bath B for 2 min.

### 3.2. Morphologies of the Inorganic Layer

**Figure 3:**
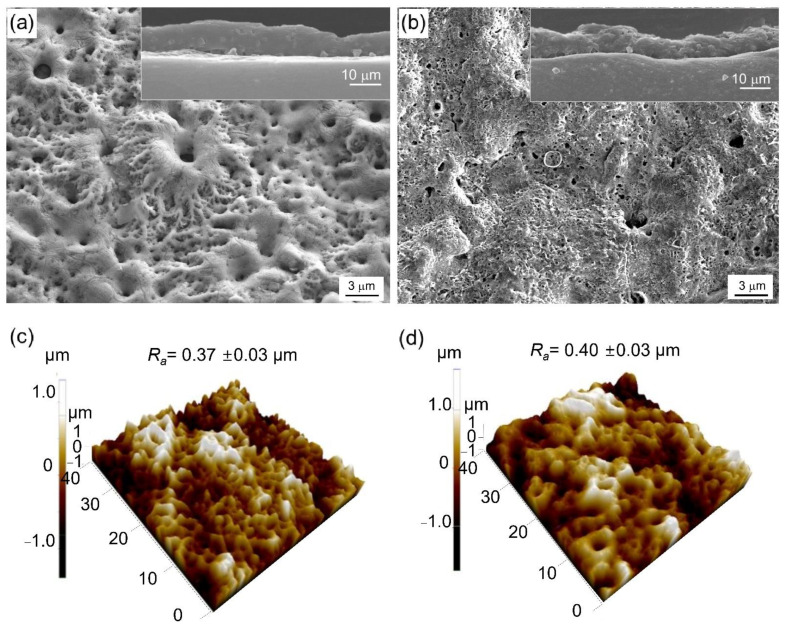
SEM images showing the surface morphologies of the oxide layer formed in (**a**) bath A and (**b**) bath B, respectively, with their cross-section as an inset. AFM images showing the surface morphologies of the present samples treated in (**c**) bath A for 10 min and (**d**) bath B for 2 min, respectively.

Figure 3a,b show surface morphologies of the samples from bath A and bath B, respectively, with the cross-sectional images as an inset. Irrespective of the electrolyte concentration, both microstructures showed a typical porous surface of the PEO layer. The oxide layer in bath B showed comparable thickness with the bath A sample, despite its shorter processing time, implying lower electricity consumption. Cross-section images of the oxide layer grown in baths A and B revealed that the average coating thicknesses were ~10 ± 0.5 µm and ~10 ± 0.2 µm, respectively. A comparable coating thickness was designed during the optimization process in order to give more clarity on the factors that might alter the properties of the oxide layer. As documented earlier, a higher coating thickness would likely to yield better corrosion protection [17,20]. Thus, by forming the same coating thickness, it can be excluded from the factors associated with performance improvement.

Figure 3c,d show the surface roughness of the samples treated in bath A and bath B for 10 min and 2 min, respectively. The surface roughness (*R_a_*) was found to be similar, with *R_a_* values of ~0.37 ± 0.03 µm and 0.40 ± 0.03 µm for bath A and bath B, respectively. In a typical PEO coating, the surface roughness increases with the increase of coating time due to the increased difference in height between the crater- and hill-like microstructures as a fingerprint of plasma discharges during PEO. In the present case, the *R_a_* values were nearly identical despite different coating times, implying that the deposition of coating components from the electrolyte species and ionized metal substrate was more efficient in the case of bath B.

### 3.3. Compositional Analysis of the Inorganic Layer

**Figure 4:**
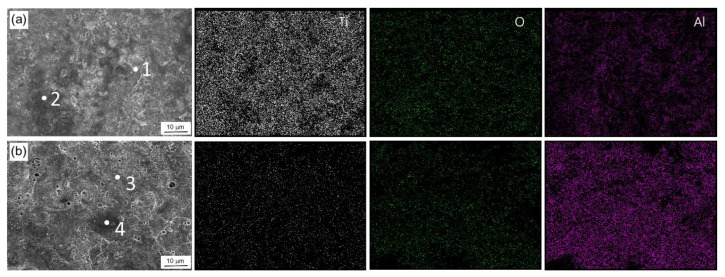
EDS map area of Ti, O, and Al elements on the surface of bath A and bath B samples after PEO for 10 min and 2 min, respectively. Points 1–4 are used for EDS point analysis, and the results are displayed in Table 1.

Figure 4a,b show a surface elemental analysis of the sample after PEO on bath A and bath B, respectively. It is shown that the intensity of Ti was clearer in the EDS mapping of sample A, whilst Al intensity was more obvious in the bath B samples, implying that a high number of Al species were incorporated. A point analysis on area 1–4 showed that the inclusion of Al-containing compounds on the surface of the samples was made to a higher extent in sample B than sample A as listed in Table 1. The fraction of Al was twice that of Ti, which might be associated with certain stoichiometric formulas of Al-containing compounds.

**Figure 5:**
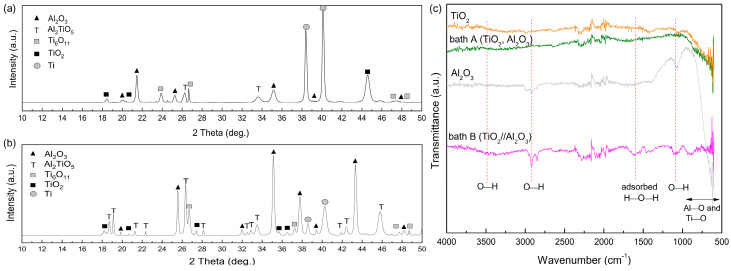
XRD pattern of samples after PEO in (**a**) bath A and (**b**) bath B, respectively. The bath B sample showed a higher amount of Al_2_TiO_5_, presumably due to the highly concentrated aluminate electrolyte. (**c**) FT-IR spectra showed peaks associated with metal-oxide bonding, O–H stretching, and water molecules.

To determine the constitutive compounds present in both samples, XRD patterns in the range of 10~50 2θ degrees are shown in Figure 5a,b. The majority of the peaks were due to the presence of TiO_2_, Al_2_O_3_, Ti_6_O_11_ and Al_2_TiO_5_. A peak for Ti was detected due to the deep penetration of X-rays during analysis. The detection of Al_2_TiO_5_ was parallel with EDS point analysis. Interestingly, with an increase of aluminate concentration in bath B, the peak of Al_2_TiO_5_ increased considerably. This fact was validated by the FT-IR analysis shown in Figure 5c. The FT-IR spectra showed peaks of metal-oxide bonding, O–H bonding, and chemisorbed water molecules from bath A and bath B samples, whereas pure Al_2_O_3_ and pure TiO_2_ were added as references. In Figure 5c, the broad peak at 3400~3500 might be associated to the O–H stretching of hydroxyl groups, while that at 1040~1090 correlated with the bending mode of hydroxyl groups. A peak with a center at 1644 indicated absorbed water molecules as well. The peaks located at 750~850 and 450~850 could be attributed to Ti–O bands and tetrahedral coordination of Al–O units [21,22]. In the present case, it is likely that these two peaks associated with Al_2_TiO_5_ overlapped with each other, indicating the presence of alumina and TiO_2_. Such a case was proven by the existence of the peaks that emerged at 600~750 and 870, which were attributed to the octahedral Al–O units [AlO_6_]^9−^ and Ti in octahedral [TiO_6_]^8−^ units [22,23]. The successful formation of Al_2_TiO_5_ correlated with a sufficient number of aluminate species during PEO which were further incorporated into the oxide layer. The incorporation of Al_2_O_3_ itself might be followed by chemical reaction under a high plasma temperature, predicted to be ~9000 K [24].

### 3.4. Corrosion Protection Capabilities of the Oxide Layer

**Figure 6:**
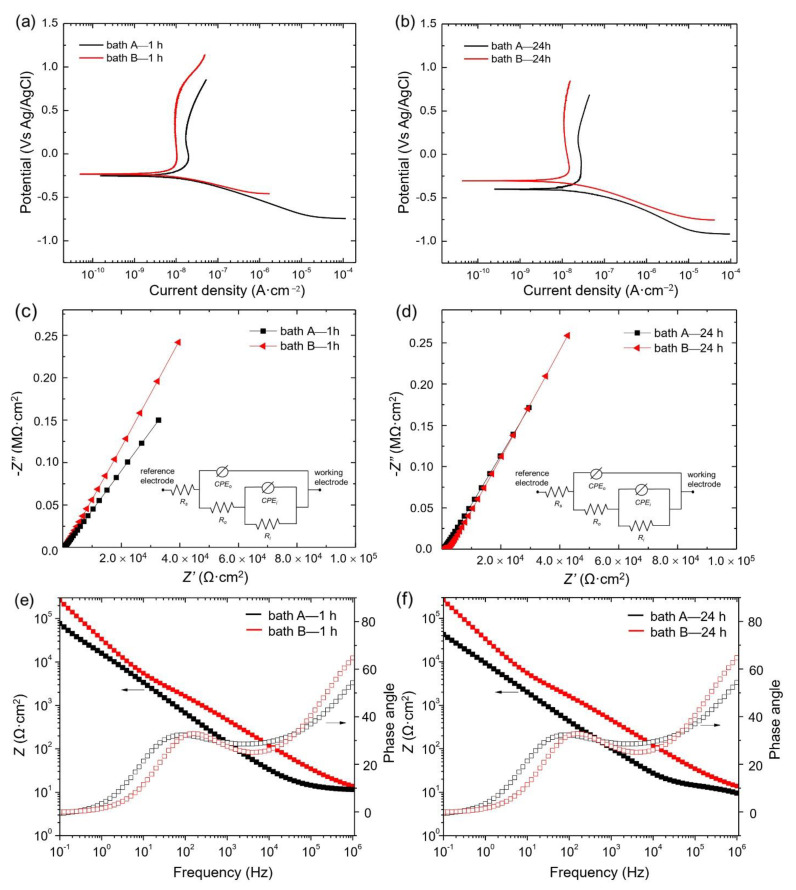
(**a**,**b**) Potentiodynamic polarization curves of the PEO–treated samples after immersion in 3.5 wt.% NaCl for 1 h, 24 h, respectively. The bath B sample showed better corrosion protection in both short and long corrosion tests. (**c**,**d**) EIS Nyquist plots and (**e**,**f**) Bode plots of all samples measured from 10^6^ to 0.1 Hz. The values were acquired based on the equivalent circuit model, shown as insets in (**c**,**d**).

The polarization curves of the oxide layer in 3.5 wt.% NaCl are shown in Figure 6a,b for 1 h and 24 h immersion duration, respectively. The electrochemical parameters of corrosion current density (*i_corr_*), corrosion potential (*E_corr_*), and cathodic Tafel slope (*β_c_*) are presented in Table 2. The *β_c_* is listed in Table 2, since only cathodic polarization was used for the calculation of (*i_corr_*) [25] according to Equation (2), whilst anodic Tafel slope (*β_a_*) could be excluded due to the lack of necessary qualities.

(2) $$R_{p}=\frac{\beta_{a}\cdot \beta_{c}}{2.303\;i_{corr}(\beta_{a}+\beta_{c})}$$

At both short and long immersion times, the corrosion resistance of bath B sample, which was processed in concentrated electrolyte, showed better corrosion resistance than the bath A sample. In particular, the corrosion rate for bath B was ~4 times slower than that of bath A at a shorter processing time, whilst at longer immersion time, it was twice as slow as that of bath A. On the other hand, the corrosion potential of bath B was found to be identical at short immersion time and shifted to a lower potential region at a longer immersion time, probably due to the formation of corrosion debris having lower *E_corr_* values [1,20].

Corrosion performance is primarily defined by how well the oxide layer isolates corrosive ions from the underlying substance. Thus, coating thickness is the main factor responsible for corrosion performance [1]. In the present case, however, due to the similar coating thicknesses and microstructural features of the oxide layer, the reason for the improvement of corrosion resistance was associated with the chemical composition of the oxide layer, specifically, the high fraction of Al_2_TiO_5_.

As a rapid and non-destructive technique, electrochemical impedance spectroscopy (EIS) provides Faradaic resistance and capacitance values related to corrosion phenomena. The equivalent circuit model shown in the inset of Figure 6c,d was well fitted to the EIS data. In this circuit, *R_s_* defines the resistance of a NaCl solution, R*_o_* the resistance of the outer layer, and R*_i_* the resistance of the inner layer of the sample. *CPE_o_* and *CPE_i_* define the constant phase elements associated with the outer and inner layers, respectively. C is associated to the capacitance of the oxide layer.

(3) $$Z_{CPE}=\frac{1}{\left[Y{\left(j\omega \right)}^{n}\right]}$$

where *ω* is the angular frequency, *j* is the imaginary number, and *Y* and *n* are *CPE* parameters. The values of *n* ranged from 0 to 1; for *n* = 0, CPE was a pure resistor [26]. In contrast, CPE serves as a pure capacitor when *n* = 1. These parameters describe the insulation tendency that occurred due to the non-uniform current distribution on the electrolyte/coating interface, where *n* reflected the degree of surface homogeneity [20,27,28,29,30].

As shown in Figure 6c,d, similar Nyquist plots could be observed from the samples, regardless of the electrolyte composition. The curves of B samples in both short and long immersion time, however, were apparently larger than those of the A sample, indicating that a smaller number of electrons passed through the electrode-electrolyte interface on the B samples, possibly due to its dense structure. Figure 6c shows the impedance of the samples from bath A and bath B, measured after a short processing time. In general, the impedance of the samples from bath A showed higher impedance, probably due to the compact characteristics of the coating. Interestingly, the bath B sample showed the highest impedance among the samples, probably due to the thick and compact coating as a result of the high uptake of aluminate species during PEO. *R_i_* was found to be higher than *R_o_*, irrespective of the electrolyte composition. This phenomenon suggested that the inner layer-substrate interface was responsible primarily for protecting the substrate from corrosion during the corrosion test. The details of electrochemical responses can be seen in Table 3.

The protective characteristics could be determined using |Z|_0.1Hz_ in the Bode impedance plot, where a greater value indicates a reduced rate of corrosion. As can be seen in Figure 6e,f, the values of |Z|_0.1Hz_ for the bath B sample were higher than those of the other samples at both low and high frequency ranges. The low frequency range was attributed to the inner part of the oxide layer, whilst the high frequency range was attributed to the outer porous layer. In general, the inner layer showed higher impedance as compared to the outer layer, owing to its relatively dense structure. From the plots of the Bode phase angle shown in Figure 6e,f, two constant phase elements were identified in both samples; these were related to the response of the outer and inner parts of the oxide layer.

The EIS curves might exhibit several time-constants depending on whether the number of layers have distinct coating features, whether they are dense or porous, whether a space charge region was formed, blocking the charge transfer, or whether the charge transfer was affected by diffusion limitation [31]. As shown in the phase-angle plots in Figure 6e,f, at least two-time constants were identified from both samples. It is also evident that the bath B samples had higher phase angle values, corresponding to a higher frequency (10^6^ Hz) as compared to the bath A counterpart, suggesting higher corrosion protection performance. It is worth noting that the impedances and phase angles shown in Figure 6e-f showed similar tendencies. In general, the impedance response for low frequency was higher than that of high frequency, which indicated that the inner layer exhibited better corrosion resistance as compared to the outer layer for both samples. With careful observation, however, the impedance of bath A sample at lower frequency decreased to some extent with prolonged immersion time from 1 to 24 h, which implied that the resistance of the inner part of the oxide layer had decreased considerably. On the other hand, the impedance response from higher frequency showed no alteration, which indicated that the extent of the corrosion progress in the outer layer region was suppressed. Such phenomena might indicate that the corrosive ions which infiltrated the outer part of the oxide layer had reached and caused local corrosion in the inner layer. In contrast, the impedance values of both inner and outer parts of the oxide layer in bath B were found to be consistent, implying that the overall oxide layer was relatively stable during the immersion period of 1 to 24 h. It is noteworthy that since EIS analysis is a non-destructive method with the use of a small magnitude of alternating current during the test, the extent of corrosion might show slower tendencies as compared to that of potentiodynamic polarization test [26,31].

In addition, a deviation of the phase maximum values away from 90 degrees indicated a non-ideal capacitive behavior. Lopez-Ortega et al. [32] suggested that this phenomenon might arise from inhomogeneities in the dielectric properties of the oxide layer, such as porosity, mass transport, and the relaxation effect. In many cases, the time constants would be difficult to observe due to an overlying constraint [33,34]. On the other hand, the phase angle values at high frequencies close to 0° were characteristic of purely resistive behavior [35,36].

### 3.5. Photocatalytic Activity

**Figure 7:**
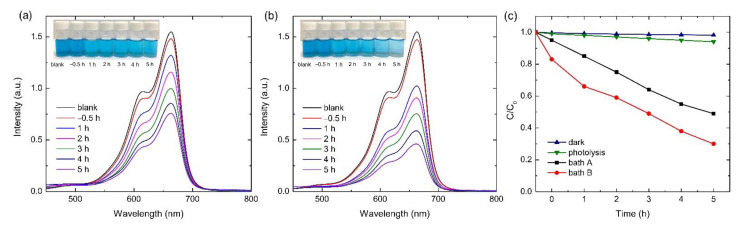
Curves showing photocatalytic properties (PA) of (**a**) bath A and (**b**) bath B samples for degradation of methylene blue (MB) under UV light irradiation for −0.5 to 5 h. Inset represents photographic observation of MB solution after catalysis. (**c**) Degradation kinetic curve of the oxide layer developed by PEO in bath A and bath B under UV light irradiation for −0.5 to 5 h. The catalytic degradation of MB under a dark environment without UV irradiation (dark) and under UV irradiation with the absence of samples (photolysis) were added as a reference.

The photocatalysis activity (PA) was observed from the degradation of methylene blue (MB) under UV light irradiation in a time range of −0.5 to 5 h with blank sample as a comparison. The effect of adsorption was studied by immersing the sample in MB solution for 30 min prior to UV irradiation, which was then annotated as adsorption at −0.5 h. Figure 7a,b show the degradation of MB on the surface of bath A and bath B samples. The PA of the bath B sample showed a higher value than that of the bath A sample, presumably due to the different surface composition of the oxide layers, since the microstructural features of both samples showed a high degree of similarity. On the other hand, the factor coming from the adsorption reaction was less obvious from the curve, implying that the chemical degradation was more dominant as compared to the adsorption process. The photocatalytic activities were summarized by plotting the concentration ratio of MB to the blank solution with respect to coating time, as depicted in Figure 7c. From Figure 7c, it can be seen that bath A showed low photocatalytic degradation; only ~50% MB was decomposed after 5 h. Interestingly, the same extent of degradation took place at shorter time in the bath B sample, i.e., about 3 h under UV irradiation. Upon the completion of 5 h UV irradiation, 70% of MB was found to be decomposed. This 20% improvement in photocatalytic efficiency could be meaningful for industrial applications. In addition, the degradation of MB for the bath B sample (representative) was recorded to be negligible when conducted in a dark condition (without UV irradiation). On the other hand, the photolysis curve of MB under UV irradiation (without sample) showed a low degree of degradation, i.e., less than 5%. Such phenomena confirmed that the main mechanism for such degradation was photocatalysis, where UV light was mandatory to induce the catalytic reaction for the degradation of dyes by active compounds.

Given that all PEO coatings had nearly equal morphologies, thicknesses, and constituent compounds, the PA values were associated with the different contents of Al_2_TiO_5_ incorporated into TiO_2_/Al_2_O_3_ oxide layer, as governed by the concentration of Al_2_O_3_ particles in the supporting electrolyte. The Al_2_O_3_ impurity might alter the band gap of TiO_2_, which would result in a change of photocatalytic activity. Al_2_O_3_ particles contribute to the PA primarily by expanding the optical absorption range of TiO_2_/Al_2_O_3_ coatings or by preventing fast recombination of photo-generated electron/hole pairs through the narrowing band gap of the TiO_2_ in the oxide layer. The band gap of Al_2_TiO_5_ was found to be 2.88 eV [37], smaller than that of TiO_2_ (3.2 eV) [38,39], which might have further contributed to the decrease in the band gap between of the oxide layer. A narrow band gap leads to the improvement of photocatalytic properties by decreasing the overall value of band gap energy and shifting the absorption edge of TiO_2_ to the visible light region [40]. A previous investigation on the development of an Al_2_TiO_5_ via sol-gel method reported that such a compound can trigger an effective separation of photo-induced charge carriers and achieve high thermal stability [41,42]. On the other hand, several factors that might affect the photocatalytic properties of photo-active materials have been documented, including the increase of surface area, decoration of the surface with noble metal particles (Ag, Au, Pd), and the formation of constituent compounds with lower electron-hole recombination rates, such as anatase (over rutile) [37,38,39,40,41,42].

The present investigation suggested that a combination of protective capabilities and catalytic performance could be achieved through the use of highly concentrated aluminate solution via PEO on pure Ti. Since the results are qualitative in nature, further study is required to develop a robust foundation based on a detailed quantitative approach to understand the relationship between the concentration of the electrolyte and the constituent compounds, as well as the microstructural characteristics of the oxide layer.

## 4. Conclusions

A combination of both protective and functional layers was achieved via plasma electrolytic oxidation on a pure titanium alloy by employing a highly concentrated electrolyte with a short processing time. The high corrosion resistance arose from the comparable thickness of the oxide layer processed at longer duration, whilst active photocatalytic performance was attributed to the high fraction of Al_2_TiO_5_, which is known to have a low band gap. The formation of Al_2_TiO_5_ via PEO was relatively faster than in methods documented by other research groups.

## Figures and Tables

**Table 1:** EDS results of the PEO-treated Cp-Ti in two different aluminate concentrations: low concentration (bath A) and high concentration (bath B). Points for analysis are taken from the surface area shown in Figure 4.

Sample	Point	Ti (wt.%)	O (wt.%)	Al (wt.%)
bath A	1	50.16	43.30	6.54
	2	50.40	43.39	6.21
bath B	3	31.26	14.31	54.42
	4	27.26	32.1	40.03

**Table 2:** Polarization resistance of the PEO-treated Cp-Ti in two different aluminate concentrations: low concentration (bath A) and high concentration (bath B). The polarization tests were performed in 3.5 wt.% NaCl solution measured from −0.25 to 0.4 V vs. open circuit potential.

Sample	*i_corr_* (A·cm^2^)	*E_corr_* (V)	*β_c_* (V/Decade)
bath A—1 h	4.18 × 10^−8^	−0.26	−0.16
bath B—1 h	9.87 × 10^−9^	−0.23	−0.12
bath A—24 h	2.82 × 10^−8^	−0.40	−0.16
bath B—24 h	1.43 × 10^−8^	−0.29	−0.15

**Table 3:** Electrochemical impedance parameters of the PEO-treated Cp-Ti at two different aluminate concentrations: low concentration (bath A) and high concentration (bath B). The impedance tests were performed in simulated seawater (3.5 wt.% NaCl solution) under a frequency range of 10^6^ to 0.1 Hz. With the equivalent circuit model displayed in the inset of Figure 6c–d, the values were produced iteratively.

Sample	R_s_ (Ω·cm^2^)	*R_o_* (Ω·cm^2^)	*R_i_* (Ω·cm^2^)	*n_o_*	*CPE_o_* (S·s^n^·cm^−2^)	*n_i_*	*CPE_i_* (S·s^n^·cm^−2^)
bath A—1 h	8.99	3.81 × 10^1^	3.01 × 10^6^	0.93	2.30 × 10^−5^	0.51	2.30 × 10^−5^
bath B—1 h	4.65	1.11 × 10^−2^	7.72 × 10^4^	0.82	2.94 × 10^−6^	0.47	4.94 × 10^−5^
bath A—24 h	2.17	1.84 × 10^3^	2.23 × 10^4^	0.68	6.10 × 10^−6^	0.55	1.57 × 10^−5^
bath B—24 h	4.31	3.32 × 10^1^	3.16 × 10^4^	0.79	4.08 × 10^−6^	0.39	1.19 × 10^−4^

## Data Availability

Not applicable.

## References

[1] Kaseem M., Fatimah S., Nashrah N., Ko Y.G. (2021). Recent progress in surface modification of metals coated by plasma electrolytic oxidation: Principle, structure, and performance. Prog. Mater. Sci..

[2] Zakaria A., Shukor H., Todoh M., Jusoff K. (2020). Bio-functional coating on Ti_6_Al_4_V surface produced by using plasma electrolytic oxidation. Metals.

[3] Kaseem M., Choi K., Ko Y.G. (2017). A highly compact coating responsible for enhancing corrosion properties of Al-Mg-Si alloy. Mater. Lett..

[4] Peters M., Hemptenmacher J., Kumpfert J., Leyens C. (2003). Titanium and Titanium Alloys, Fundamentals and Applications, Chapter 1—Structure and Properties of Titanium and Titanium Alloys.

[5] Shin K.R., Ko Y.G., Shin D.H. (2012). Surface characteristics of ZrO_2_-containing oxide layer in titanium by plasma electrolytic oxidation in K4P_2_O_7_ electrolyte. J. Alloys Compd..

[6] Yerokhin A.L., Nie X., Leyland A., Matthews A., Dowey S.J. (1999). Plasma electrolysis for surface engineering. Surf. Coat. Technol..

[7] Jung Y.C., Shin K.R., Ko Y.G., Shin D.H. (2014). Surface characteristics and biological response of titanium oxide layer formed via micro-arc oxidation in K3PO4 and Na3PO4 electrolytes. J. Alloys Compd..

[8] Lee K.M., Ko Y.G., Shin D.H. (2011). Incorporation of carbon nanotubes into micro-coatings film formed on aluminum alloy via plasma electrolytic oxidation. Mater. Lett..

[9] Kaseem M., Ko Y.G. (2019). Morphological modification and corrosion response of MgO and Mg_3_(PO_4_)_2_ composite formed on magnesium alloy. Compos. Part B Eng..

[10] Molaei M., Fattah-Alhosseini A., Gashti S.O. (2018). Sodium Aluminate Concentration Effects on Microstructure and Corrosion Behavior of the Plasma Electrolytic Oxidation Coatings on Pure Titanium. Metall. Mater. Trans. A Phys. Metall. Mater. Sci..

[11] Cheng Y.L., Cao J.H., Mao M.K., Peng Z.M., Skeldon P., Thompson G.E. (2015). High growth rate, wear resistant coatings on an Al-Cu-Li alloy by plasma electrolytic oxidation in concentrated aluminate electrolytes. Surf. Coat. Technol..

[12] Yang W., Li Q., Liu W., Liang J., Peng Z., Liu B. (2017). Characterization and properties of plasma electrolytic oxidation coating on low carbon steel fabricated from aluminate electrolyte. Vacuum.

[13] Li R., Li T., Zhou Q. (2020). Impact of Titanium Dioxide (TiO_2_) Modification on Its Application to Pollution Treatment—A Review. Catalyst.

[14] Utu I.D., Marginean G., Hulka I., Serban V.A., Cristea D. (2015). Properties of the thermally sprayed Al_2_O_3_-TiO_2_ coatings deposited on titanium substrate. Int. J. Refract. Met. Hard Mater..

[15] Zoubi W.A., Al-Hamdani A.A.S., Sunghun B., Ko Y.G. (2021). A review on TiO_2_-based composites for superior photocatalytic activity. Rev. Inorg. Chem..

[16] Fujishima A., Rao T.N., Tryk D.A. (2000). Titanium dioxide photocatalysis. J. Photochem. Photobiol. C Photochem. Rev..

[17] Fatimah S., Kamil M.P., Kwon J.H., Kaseem M., Ko Y.G. (2017). Dual incorporation of SiO_2_ and ZrO_2_ nanoparticles into the oxide layer on 6061 Al alloy via plasma electrolytic oxidation: Coating structure and corrosion properties. J. Alloys Compd..

[18] Shin K.R., Ko Y.G., Shin D.H. (2011). Effect of electrolyte on surface properties of pure titanium coated by plasma electrolytic oxidation. J. Alloys Compd..

[19] Ikonopisov S. (1977). Theory of electrical breakdown during formation of barrier anodic films. Electrochim. Acta.

[20] Kamil M.P., Kaseem M., Ko Y.G. (2017). Soft plasma electrolysis with complex ions for optimizing electrochemical performance. Sci. Rep..

[21] Habibi S., Jamshidi M. (2020). Materials Science in Semiconductor Processing Synthesis of TiO_2_ nanoparticles coated on cellulose nanofibers with different morphologies: Effect of the template and sol-gel parameters. Mater. Sci. Semicond. Process..

[22] Stanciu L.A., Groza J.R., Jitianu A., Zaharescu M., Stanciu L.A., Groza J.R., Jitianu A., Zaharescu M. (2004). Structural Evolution During Reaction to Form Aluminum Titanate from Sol-Gel Precursors. Mater. Manuf. Process..

[23] Sharma M., Singh D.K., Upadhyay R.K., Yadav M.S., Amritphale S.S., Chandra N. (2014). Novel approach for sol-gel synthesis of nanosize aluminium titanate. Mater. Res. Innov..

[24] Hussein R.O., Northwood D.O., Nie X. (2010). Coating growth behavior during the plasma electrolytic oxidation process. J. Vac. Sci. Technol. A Vac. Surf. Film..

[25] Jayaraj J., Amruth Raj S., Srinivasan A., Ananthakumar S., Pillai U.T.S., Dhaipule N.G.K., Mudali U.K. (2016). Composite magnesium phosphate coatings for improved corrosion resistance of magnesium AZ31 alloy. Corros. Sci..

[26] Kaseem M., Kamil M.P., Kwon J.H., Ko Y.G. (2015). Effect of sodium benzoate on corrosion behavior of 6061 Al alloy processed by plasma electrolytic oxidation. Surf. Coat. Technol..

[27] Ebrahimi N., Momeni M., Kosari A., Zakeri M., Moayed M.H. (2012). A comparative study of critical pitting temperature (CPT) of stainless steels by electrochemical impedance spectroscopy (EIS), potentiodynamic and potentiostatic techniques. Corros. Sci..

[28] Isakhani-Zakaria M., Allahkaram S.R., Ramezani-Varzaneh H.A. (2019). Evaluation of corrosion behaviour of Pb-Co_3_O_4_ electrodeposited coating using EIS method. Corros. Sci..

[29] Venkateswarlu K., Rameshbabu N., Sreekanth S., Bose A.C., Muthupandi V., Babu N.K., Subramanian S. (2012). Role of electrolyte additives on in-vitro electrochemical behavior of micro arc oxidized titania films on Cp Ti. Appl. Surf. Sci..

[30] Zhao X., Zuo Y., Zhao J., Xiong J., Tang Y. (2006). A study on the self-sealing process of anodic films on aluminum by EIS. Surf. Coat. Technol..

[31] Gnedenkov S.V., Sinebryukhov S.L., Sergienko V.I. (2006). Electrochemical impedance simulation of a metal oxide heterostructure/ electrolyte interface: A review. Russ. J. Electrochem..

[32] López-Ortega A., Arana J.L., Rodríguez E., Bayón R. (2018). Corrosion, wear and tribocorrosion performance of a thermally sprayed aluminum coating modified by plasma electrolytic oxidation technique for offshore submerged components protection. Corros. Sci..

[33] Mohedano M., Blawert C., Zheludkevich M.L. (2015). Silicate-based Plasma Electrolytic Oxidation (PEO) coatings with incorporated CeO_2_ particles on AM50 magnesium alloy. Mater. Des..

[34] Mingo B., Arrabal R., Mohedano M., Llamazares Y., Matykina E., Yerokhin A., Pardo A. (2018). Influence of sealing post-treatments on the corrosion resistance of PEO coated AZ91 magnesium alloy. Appl. Surf. Sci..

[35] Imaz N., Ostra M., Vidal M., Díez J.A., Sarret M., García-lecina E. (2014). Corrosion behaviour of chromium coatings obtained by direct and reverse pulse plating electrodeposition in NaCl aqueous solution. Corros. Sci..

[36] Heakal F.E., Tantawy N.S., Shehta O.S. (2011). Influence of chloride ion concentration on the corrosion behavior of Al-bearing TRIP steels. Mater. Chem. Phys..

[37] Azarniya A., Soltaninejad M., Zekavat M., Bakhshandeh F., Madaah Hosseini H.R., Amutha C., Ramakrishna S. (2020). Application of nanostructured aluminium titanate (Al_2_TiO_5_) photocatalyst for removal of organic pollutants from water: Influencing factors and kinetic study. Mater. Chem. Phys..

[38] Khaki M.R.D., Shafeeyan M.S., Raman A.A.A., Daud W.M.A.W. (2017). Application of doped photocatalysts for organic pollutant degradation—A review. J. Environ. Manage..

[39] Khan M.M., Ansari S.A., Pradhan D., Ansari M.O., Han D.H., Lee J., Cho M.H. (2014). Band gap engineered TiO_2_ nanoparticles for visible light induced photoelectrochemical and photocatalytic studies. J. Mater. Chem. A.

[40] Bayati M.R., Moshfegh A.Z., Golestani-Fard F. (2010). Synthesis of narrow band gap (V_2_O_5_)x-(TiO_2_) 1-x nano-structured layers via micro arc oxidation. Appl. Surf. Sci..

[41] Bakhshandeh F., Azarniya A., Madaah Hosseini H.R., Jafari S. (2018). Are aluminium titanate-based nanostructures new photocatalytic materials? Possibilities and perspectives. J. Photochem. Photobiol. A Chem..

[42] Azarniya A., Zekavat M., Soltaninejad M., Bakhshandeh F., Khatiboleslam S., Ramakrishna S. (2020). Preparation of nitrogen-doped aluminium titanate (Al_2_TiO_5_) nanostructures: Application to removal of organic pollutants from aqueous media. Adv. Powder Technol..

